# Raising fluid walls around living cells

**DOI:** 10.1126/sciadv.aav8002

**Published:** 2019-06-05

**Authors:** Cristian Soitu, Alexander Feuerborn, Cyril Deroy, Alfonso A. Castrejón-Pita, Peter R. Cook, Edmond J. Walsh

**Affiliations:** 1 Oxford Thermofluids Institute, Department of Engineering Science, University of Oxford, Osney Mead, Oxford OX2 0ES, UK.; 2 The Sir William Dunn School of Pathology, University of Oxford, South Parks Road, Oxford OX1 3RE, UK.; 3 Iota Sciences Ltd., Begbroke Science Park, Begbroke, Oxfordshire OX5 1PF, UK.; 4 Department of Engineering Science, University of Oxford, Parks Road, Oxford, OX1 3PJ, UK.

## Abstract

An effective transformation of the cell culture dishes that biologists use every day into microfluidic devices would open many avenues for miniaturizing cell-based workflows. In this article, we report a simple method for creating microfluidic arrangements around cells already growing on the surface of standard petri dishes, using the interface between immiscible fluids as a “building material.” Conventional dishes are repurposed into sophisticated microfluidic devices by reshaping, on demand, the fluid structures around living cells. Moreover, these microfluidic arrangements can be further reconfigured during experiments, which is impossible with most existing microfluidic platforms. The method is demonstrated using workflows involving cell cloning, the selection of a particular clone from among others in a dish, drug treatments, and wound healing. The versatility of the approach and its biologically friendly aspects may hasten uptake by biologists of microfluidics, so the technology finally fulfills its potential.

## Teaser

Microfluidics with fluid walls opens up the realm of reconfiguring cellular environments during assays.

## INTRODUCTION

Microfluidic-based approaches have been applied to many workflows in biology; however, uptake by mainstream biology laboratories remains slow. Many reasons for this have been given, including the facts that the materials used are not congenial to cell growth; architectures are enclosed and thus inaccessible; geometries are predetermined and cannot be reconfigured during experiments; costs of manufacture and operation are high; and the workflows typically designed by engineers often do not align with the preexisting ones that biologists have spent decades developing (*1*–*3*).

Open microfluidic approaches have sought to address some of these issues through their low barriers to fabrication and operation. However, such platforms often require established live-cell–based assays to be compatible with, or adapted to, materials and methods not traditionally used in biology, as is the case with suspended (*4*), in-air (*5*), and paper-based microfluidics (*6*). Another open approach uses inserts to form hydrogel walls within wells of standard microplates, so that the substrates familiar to biologists can be repurposed for microfluidics (*7*). However, these methods have been demonstrated by creating microfluidic arrangements before incorporating cells. A more biologically relevant approach would be to form arrangements on petri dishes in which biologists traditionally grow their cells (that is, begin with what biologists already use and fabricate the required microfluidic devices on their preexisting platform).

In the macroworld, solid materials are used as the building blocks for most objects, and the idea of creating structures with liquid walls does not work—gravity collapses them into puddles. Therefore, liquids are held in chambers with solid walls at the macroscale and in most microfluidic devices. However, as length scales become smaller, interfacial forces dominate gravitational ones (i.e., small Bond numbers) and confinement of liquids by fluid interfaces, analogous to solid materials, becomes possible; therefore, liquid walls can replace solid ones. While the creation of three-dimensional (3D) constructs with fluid walls has been previously demonstrated using nanoparticle surfactants, the biocompatibility of the system remains to be assessed (*8*). Recently, we developed a method for making arrays of isolated microfluidic chambers on virgin petri dishes to accommodate some major workflows used in cell biology [e.g., cell feeding and transfer, cloning, cryopreservation, fixation and immunolabeling, cell lysis and reverse transcription polymerase chain reaction (RT-PCR), and CRISPR-Cas9 gene editing]; in these cases, cells were added after fabrication (*9*). Here, we create various microfluidic arrangements on standard petri dishes already containing adherent cells and go on to reconfigure them in real time. Moreover, we isolate and retrieve cell clones and perform proof-of-concept drug tests and wound-healing assays. The ability to create and reconfigure microfluidic circuits on petri dishes as cells grow and divide should find many applications in biology and yield more relevant phenotypic and genotypic responses when compared to standard microfluidic assays.

## RESULTS

### Methodology

**Fig. 1:**
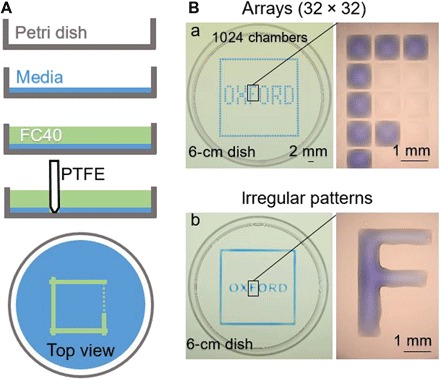
Chamber construction. (**A**) Principle. Dulbecco’s modified Eagle’s medium (DMEM) + 10% fetal bovine serum (FBS) is added to a virgin petri dish, and most of the medium is removed to leave a thin film covering the bottom, which is overlaid with FC40. The stylus is moved across the bottom to create a microfluidic arrangement. When complete, the initial volume of DMEM + 10% FBS will be divided into two parts separated by a continuous liquid wall of FC40 pinned to the substrate. (**B**) Different patterns. (a) Forming equally spaced vertical and horizontal lines creates an array (32 × 32; 1-mm spacing). Next, 60 nl of blue dye is added by the printer to selected chambers; peripheral chambers receive blue dye to give the blue square, and internal ones give the word “OXFORD.” The magnification (right) shows individual chambers without and with dye. (b) A similar pattern is created by forming two squares (one slightly larger than the other) with the stylus and then adding dye manually to the space in between; each letter is made by forming its sides and again manually filling the interior. The magnification shows that the letter “F” is one continuous body of liquid. Photo credit: Cristian Soitu, University of Oxford.

Figure 1A illustrates the approach; fluids sitting in a standard petri dish are reshaped to create the desired microfluidic arrangement. In summary, the bottom of a virgin petri dish is covered with tissue culture medium, and most of the medium is removed to leave a thin film covering the polystyrene substrate. The thin film is overlaid with an immiscible fluorocarbon, FC40, to prevent evaporation; this additionally provides a physical barrier against external contaminants and thus maintains sterility of the medium. A hydrophobic and fluorophilic stylus [which has a conical tip made of polytetrafluoroethylene (PTFE), or “Teflon”] is lowered through the two liquid layers to bring it into contact with the bottom of the dish. As the fluorocarbon preferentially wets Teflon (compared to water), FC40 is brought down to the substrate and displaces the aqueous phase (as it also preferentially wets the substrate). The Teflon stylus is moved laterally over the surface to create a wanted microfluidic arrangement—in this case, a square. The apparatus that draws the pattern—a “printer”—consists of a tool head that carries the stylus attached to a three-axis traverse controlled by appropriate software; the tool head also carries a dispensing tube connected through a Teflon tube to a syringe pump that can add/remove nanoliter volumes to/from any point in the microfluidic arrangement. This approach brings the advantages of open microfluidic platforms to standard cell culture ware.

The aqueous phase can be shaped in various ways, for example, by forming horizontal and vertical lines of equal length to create a grid. For instance, Fig. 1B (a) illustrates the “printing” of a 32 × 32 array of 1 mm × 1 mm chambers using tissue culture medium (which appears light pink at the macroscale due to the phenol red added as a pH indicator) and FC40 (which is colorless). The volume of medium initially in each chamber in this array is so small [i.e., ~25 nl (*9*)] that individual chambers appear colorless and invisible at low magnification. Therefore, to aid visualization, 60 nl of blue dye is added to selected chambers; each peripheral one receives blue dye (giving the blue square), as do selected ones in the interior (giving the word “OXFORD”). The amount of fluid that can be added to (or removed from) a chamber without altering its footprint depends on the advancing and receding contact angles, θ_A_ and θ_R_ (*1*, *9*).

An analogous microfluidic arrangement made by forming both curved and straight lines of various lengths is illustrated in Fig. 1B (b). Here, the outer border is made by forming two squares (one slightly larger than the other) and then adding dye to the space in between; each letter is made by forming its sides and again filling the interior.

### Reconfiguring microfluidic arrangements

**Fig. 2:**
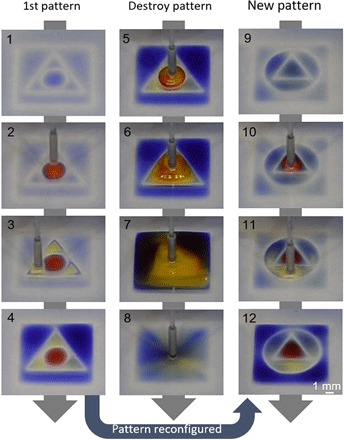
Reconfiguring microfluidic arrangements. Images show frames from movie S1. (**1**) An initial pattern is printed: a circle (radius, 1.5 mm) inside a triangle (side, 7 mm) inside a square (side, 9 mm). (**2** to **4**) Different dyes are added to each compartment (1.5 μl of red dye, 1.5 μl of yellow dye, and 5 μl of blue dye); dyes are confined within FC40 walls. (**5**) More yellow dye is added to the circle. (**6**) After adding 3 μl of yellow dye, the circular pinning line ruptures and contents spill into the triangle. (**7**) After adding 24 μl, the triangular pinning line ruptures and contents spill into the square. (**8**) Sixty microliters is withdrawn from the square. (**9**) A new pattern is printed—a triangle (side, 4.5 mm) in a circle (radius, 3.3 mm)—in the initial square. (**10** to **12**) Colored dyes are added to the three different compartments as before. Photo credit: Cristian Soitu, University of Oxford.

The method is extended to reconfigure a microfluidic arrangement during the assay; fluid walls are built, destroyed, and rebuilt in different shapes. A circle within a triangle within a square (9-mm sides) is “printed” (Fig. 2, 1; this—and following frames—is taken from movie S1), and microliter quantities of three dyes of different colors are added to the three shapes; FC40 walls prevent dyes from mixing (Fig. 2, 2 to 4). The circular wall is now destroyed by pumping yellow dye through the dispensing tube into the center (Fig. 2, 5). After adding 3 μl, θ_A_ of the circular pinning line is exceeded, the circular wall ruptures, and contents spill out into the triangle (Fig. 2, 6). After adding 24 μl, the triangular wall ruptures, and contents spill into the square (Fig. 2, 7). Now, the two internal walls are rebuilt, but this time the circle encloses a triangle. As printing through a thick aqueous film is difficult, most volume within the square is removed (Fig. 2, 8), new walls are built (Fig. 2, 9), and different colored dyes are added to the three volumes (Fig. 2, 10 to 12). These results show that FC40 walls can be destroyed and rebuilt in any desired 2D shape and that they confine liquids effectively.

### Building fluid walls around single cells and clones

**Fig. 3:**
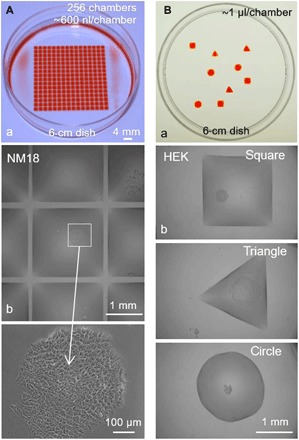
Building walls before or after cloning cells. (**A**) Building walls before plating single cells in individual chambers. After making grids (16 × 16, 2-mm spacing; 6-cm dishes), dye or cells (NM18) were added by the printer. (a) Red dye (600 nl) is added to each chamber to illustrate the grid used. (b) Medium (600 nl) containing 0.2 cells is added to each chamber (so only a few chambers receive one cell), dishes are incubated (8 days), and chambers are imaged; the central chamber contains one colony (see magnification). (**B**) Building walls around living clones. (a) Red dye (1 μl) is added to various shapes printed in a dish; the dye is added to aid visualization (see also movie S2). (b) HEK cells were plated in a dish (density, ~1 cell/cm^2^) and allowed to grow for 8 days into colonies. Next, most of the medium is removed to leave a thin film on the surface, FC40 is added, various shapes are drawn around colonies, each chamber is filled with 1 μl of medium using the printer, and images are collected. The three images come from different dishes. Photo credit: Cristian Soitu, University of Oxford

Arrays of chambers (“grids”) have been used in various workflows in cell biology; Fig. 3A recapitulates the cloning of mouse mammary tumor cells (NM18) and follows a previous workflow (*9*), where grids are initially created and cells are added later. With a 16 × 16 array (illustrated with red dye in Fig. 3A, a), a single mouse tumor cell in 600 nl is dispensed into every fifth chamber in the grid (so Poisson statistics ensure that few chambers contain two cells), and the dish is placed in a conventional CO_2_ incubator. As FC40 is freely permeable to O_2_ and CO_2_, cells grow like their counterparts lacking a fluorocarbon overlay (Fig. 3A, b). A quantitative analysis, presented in fig. S2, demonstrates that there is no significant difference between the two systems in the size of the colonies after 8 days. This confirms the biocompatibility of the platform. Moreover, when compared to imaging microplates (where solid walls around individual wells can obscure views), here, fluid walls/ceilings provide excellent optical clarity across the complete footprint of a chamber—a critical feature required to give users confidence that the chamber contains only one cell.

It is often useful to isolate one clone from others in a set of many clones (e.g., because cells in that clone express a particular phenotype or protein). Therefore, we now reverse the process: Single cells are allowed to grow into clones before surrounding them with fluid walls. Figure 3B (a) illustrates the shape of the chambers used, and Fig. 3B (b) shows the experiment (see also movie S2). Cells are plated sparsely and grown, and walls are built around resulting colonies. Each of the three shapes illustrated has different advantages (e.g., circular chambers have the most stable pinning lines for a given volume and footprint area, while triangles and rectangles can tessellate and thus use the maximum surface area). This shows that fluid walls with different 2D footprints can easily be built around living cells to provide assurance that colonies remain isolated from each other during subsequent treatment or retrieval. While there are many examples where cells are grown in confined spaces on prepatterned surfaces (*10*–*13*), these often require surface treatments before adding cells.

### Clone picking

**Fig. 4:**
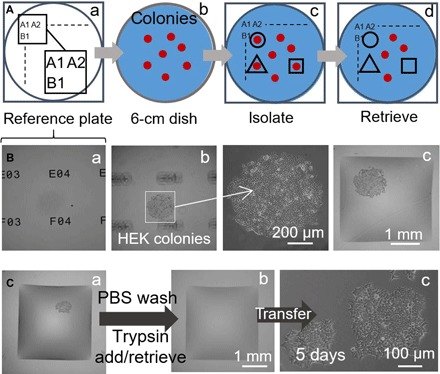
Semi-automating selective clone picking (HEK cells). The printer adds/removes a microliter to/from chambers at different stages. (**A**) Approach. (a) Locations on a glass “reference plate” are marked by unique identifiers (i.e., A1, A2 …, B1…). (b) A 6-cm dish with colonies (red) is placed on the reference plate. (c) After recording colony locations and inputting them into a script, fluid walls are printed around selected clones (black lines). (d) Clones are retrieved from these chambers. (**B**) Isolating a clone. HEK cells were plated at low density (~1 cell/cm^2^) and grown (8 days) into clones, the dish was placed on a reference plate, and walls were built around selected clones. Three different *z*-axis views of one clone are shown. (a) Reference plate with unique identifiers in focus. (b) Colony in focus (identifiers out of focus) with magnification. (c) Colony after building surrounding walls. (**C**) Clone picking. (a) Square wall built around one living colony. The printer washes cells by adding/retrieving 1 μl of PBS; it then adds 1 μl of trypsin. (b) The dish is incubated (37°C; 5 min) to detach cells from the surface, and the printer retrieves 1 μl containing the cell-rich suspension (and transfers it to a microcentrifuge tube) to leave the now-empty chamber. (c) Retrieved cells are plated manually in a 12-well microtiter plate and grown conventionally for 5 days; cells attach and grow.

We now partially automate clone picking. Our approach relies on a “reference plate,” a glass support bearing a matrix of unique identifiers (i.e., A1 and A2; Fig. 4A). After placing a dish containing colonies on this plate, clone locations are recorded using a microscope by focusing successively on the plate and clone (Fig. 4B, a and b) and inputted into a script that controls wall printing at specific positions (Fig. 4B, c). Once built, these walls effectively isolate wanted clones from others in the dish. A clone is now picked using a conventional—but miniaturized—workflow. The printer washes cells by adding/removing phosphate-buffered saline (PBS) and adds trypsin, and the dish is transferred to an incubator to enhance cell detachment; next, the printer retrieves the cell-rich suspension (emptying the chamber; Fig. 4C, a and b). Last, recovered cells are plated manually and grown conventionally; they multiply as expected (Fig. 4C, c).

### Fluid walls are effective barriers to drugs

**Fig. 5:**
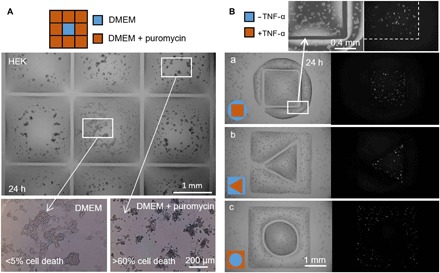
Two drug treatments side by side with untreated cells. Fluid walls were built around HEK cells (300,000 cells; 6-cm dish) grown for 24 hours. (**A**) Puromycin (3 × 3 grid; 2 mm × 2 mm chambers). The printer adds 1 μl of medium to the central chamber and 1 μl of medium + puromycin to peripheral ones (final concentration, 10 μg/ml), as indicated in the cartoon. Cell viability is assessed after incubation (37°; 24 hours) using a trypan blue exclusion assay. Cells in outer chambers are dead (more than 60% in each one), whereas those in the central one remain alive (less than 5% cell death). This assay has been replicated three times. (**B**) TNF-α. Pairs of chambers with distinct shapes are printed, one surrounding the other. The printer adds 0.5 μl of medium ± TNF-α (final concentration, 10 ng/ml) to one or other volume (as in cartoons). As cells encode a GFP-reporter gene controlled by a promoter switched on by TNF-α, they fluoresce green on exposure to the cytokine. Fluorescence images show that only cells in the treated volume fluoresce green. Volume pairs had the following dimensions: (a) square (side, 1.8 mm) in circle (radius, 1.75 mm); (b) triangle (side, 1 mm) in square (side, 3.5 mm); (c) circle (radius, 1 mm) in square (side, 3.5 mm).

We next demonstrated that fluid walls confine liquids effectively. Puromycin is a small-molecule translational inhibitor that kills mammalian cells, and is used here to demonstrate that an FC40 wall effectively prevents leakage of the poison out of square chambers (arranged as a 3 × 3 grid) drawn around cells already growing on a dish. The central chamber receives only growth medium, whereas the drug was delivered to the surrounding eight chambers at a concentration 10-fold higher than the lethal one (i.e., 10 μg/ml). After 24 hours, only cells within outer chambers die (less than 40% viability), and cells in the central one remain alive (Fig. 5A). A second example exploits a particular property of this line of human embryonic kidney (HEK) cells that had been genetically modified to encode a green fluorescent protein (GFP) reporter gene controlled by a promoter switched on by tumor necrosis factor–α (TNF-α)—when exposed to the cytokine, cells fluoresce green (*14*). Pairs of chambers with various shapes were built around cells already growing in a dish; the larger one encompasses the smaller one. As before, the flexibility of the technique is demonstrated by drawing a square inside a circle, a triangle inside a square, and a circle inside a square (Fig. 5B). After adding the cytokine to one or other chamber (inner for Fig. 5B, a and b, and outer for Fig. 5B, c), only cells in treated chambers fluoresce green. These results show that fluid walls form effective barriers to these drugs, and allow side-by-side comparison of treatments of cells growing in the same dish. It is easy to see how this approach might be extended to screen many drugs in different isolated regions of one dish.

### Wound healing

**Fig. 6:**
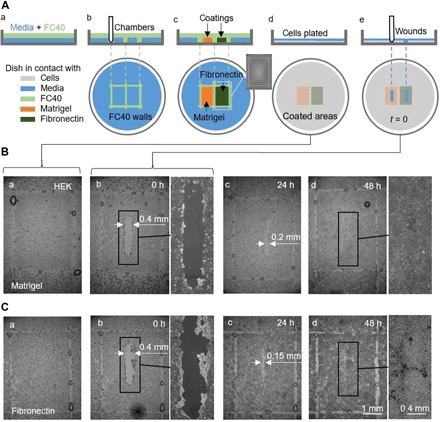
A proof-of-concept wound-healing assay using one dish precoated with Matrigel and fibronectin in different regions. (**A**) Cartoon illustrating workflow. (a) A thin layer of medium is overlaid with FC40. (b) Two chambers (3 mm × 4 mm each) are printed side by side. (c) Surfaces in chambers are coated with Matrigel or fibronectin (2 μl; final concentration of 1 μg/cm^2^; 1 hour); the inset shows an image of one chamber. Fluid walls are now destroyed, and the dish is washed with 3 ml of medium to remove unattached coatings. (d) HEK cells (600,000) are plated in the dish. (e) After 24 hours, cells have formed a monolayer, and a wound (0.4 mm × 2 mm) is created by scraping the stylus over the surface to remove cells in its path. Healing of the wound is now monitored microscopically. (**B** and **C**) Images of wounds in monolayers grown on Matrigel or fibronectin. (a and b) Immediately before and after wounding (some droplets of FC40 remain where walls originally stood). (c) After 24 hours, cell growth reduces wound widths to <0.2 mm and <0.15 mm with Matrigel and fibronectin, respectively. (d) By 48 hours, wounds have completely healed.

Wound-healing assays generally involve comparing cells growing in different conditions in different dishes (*15*–*17*), and various microfluidic devices have been developed for such assays (*18*, *19*). As many chambers can be built in one dish, and as liquid walls are easily destroyed, we exploit these features to monitor two wounds healing in one dish, in which different parts of the dish have been coated in different ways. Two coatings were used for a proof-of-concept experiment. Matrigel is a gelatinous protein mixture secreted by sarcoma cells that is often used in studies of cell invasion (*20*), and fibronectin is a glycoprotein of the extracellular matrix that enhances wound healing (*21*). The workflow (Fig. 6A) involves building two rectangular chambers and filling them with Matrigel or fibronectin to coat different parts of one surface; fluid walls are now destroyed by thorough washing, and HEK cells are plated in the dish. Cells soon form a monolayer throughout the dish except in regions originally containing walls [Fig. 6, B (a) and C (a)]; these are marked by residual FC40 droplets, and their sparser cell content presumably results from some modification of the surface. After wounding by dragging the Teflon stylus across the monolayer [Fig. 6, B (b) and C (b)], cells—now in a common liquid environment and on a common substrate that bears different coatings in different positions—migrate into the two wounds at slightly different rates (quantitative data shown in fig. S3). This workflow involves coating the surface before plating cells and wounding; it can be modified so that the coating is added later, at the time when cells begin migrating into newly formed wounds (fig. S4). It is easy to see how these workflows might be adapted to test the effects of different coatings applied at different times to arrays of wounds, each located in different regions of one petri dish; then, healing can occur in a common environment.

## DISCUSSION

A microfluidic platform for miniaturizing workflows in cell biology was recently demonstrated (*9*); it is extended here to form microfluidic arrangements around preplated adherent cells that are then used for various proof-of-principle assays (e.g., cell cloning, drug screening, and wound healing). This platform has many advantages. First, it can be used to repurpose the cell culture ware familiar to biologists into complex microfluidic arrangements (Fig. 5B) that can support elaborate workflows (Figs. 4 to 6 and fig. S4). This contrasts with conventional approaches that usually require biological assays to be adapted to suit preengineered microfluidic devices. Second, the microfluidic arrangements made using this platform have fluid walls/ceilings that are built accurately and reproducibly by interfacial forces (Figs. 1B, 2, and 3A, a). Third, the platform is highly flexible and customizable. Fourth, the replacement of solid walls with fluid ones brings all the benefits of open microfluidics without some of the limitations; for example, all points in the arrangement remain fully visible and accessible throughout an experiment because FC40 is both optically transparent and provides no barrier to a pipette tip. Fifth, the resultant pinning lines around chambers are stable (e.g., in Figs. 3 to 5 and fig. S4, fluid walls remain intact as dishes were carried between printer, CO_2_ incubator, and microscope several times). Sixth and uniquely, these microfluidic arrangements can be reconfigured in real time during an experiment (Figs. 2, 5, and 6 and fig. S4). Notable limitations include the following: Arrangements are restricted to the two dimensions of the substrate, liquid walls are inevitably more fragile than solid ones (although robust enough to withstand agitation exceeding that occurring during normal use; Figs. 1 to 6) (*9*), and the liquids and surfaces used must be matched to ensure that pinning lines are stable (e.g., some surfactants reduce volumes that may be held within chambers before pinning lines are compromised). Nevertheless, we hope that this combination of features and advantages will provide a sufficiently low barrier to entry that many biologists will begin to exploit the power of microfluidics.

## MATERIALS AND METHODS

### General reagents and equipment

FC40 was supplied by iotaSciences. All other fluids and materials were purchased from Sigma-Aldrich unless otherwise stated. Water-soluble dyes (e.g., Allura Red, toluidine blue, and resazurin) were used where indicated. Matrigel (Corning, #3542777) and fibronectin (Sigma-Aldrich, #F1141) were used for the wounding experiment.

All microfluidic arrangements were made on 6-cm polystyrene tissue culture dishes (Falcon 60 mm TC-treated Cell Culture Dish; product #353002) using custom-written software and a printer (iotaSciences Ltd., Oxford, UK). The printer is essentially a tool head driven by a three-axis traverse and appropriate software; the tool head holds a stylus—a Teflon rod (3.8 mm diameter) with a conical tip (angle at tip 50°)—and stainless steel dispensing needle (width, 0.5 mm outer diameter) connected to a syringe pump that is used to add/remove nanoliter volumes from chambers. A hydrophobic sleeve (a section of Teflon tubing) was added around the end of the dispensing needle to prevent liquids running up the outside instead of being delivered to the chamber. The stage of the printer can accommodate a 6-cm dish and microcentrifuge tubes for storing needed reagents (e.g., cell culture medium, 70% ethanol for sterilization, and tubes for waste). A tightly fitting circular sleeve around each dish acts as a positioning ring to ensure that the dish can be removed and added back on to the printer in the original orientation. The printer can be placed in a biosafety cabinet if sterility is required; then, standard procedures for sterility were used (e.g., stylus and tubes were sterilized with 70% ethanol).

### Cells

Adherent HEK cells were grown in Dulbecco’s modified Eagle’s medium (DMEM) + 10% fetal bovine serum (FBS; Gibco, Life Sciences) and subcultured using trypsin (TrypLE, Gibco, Gaithersburg, MD, #12563011). These HEK cells were genetically modified reporter cells [nuclear factor κB (NF-κB)/293/GFP-Luc Transcriptional Reporter Cell Line; System Biosciences, catalog no. TR860A-I]; they encode a GFP gene under the control of the minimal cytomegalovirus promoter downstream of four copies of the NF-κB consensus transcriptional response element, and GFP expression can be induced by TNF-α (used at a final concentration of 10 ng/ml and obtained from PeproTech, London, UK).

For Fig. 3A and fig. S2, a line of mouse mammary tumor cells (NM18) was used. To obtain clones, cells were plated at densities of 0.2 cells per well (96-well plate) and 0.2 cells per chamber (16 × 16 grid). Fluidic chambers in the grid stored 600 nl of medium, which was replenished every 3 days.

### Printing and operation of grids

DMEM + 10% FBS was used to make all grids; when the term “medium” is used, it should be assumed that serum is present. Typically, 1 ml of medium was pipetted into the dish so that the medium completely covered the bottom, the dish was tilted, ~0.9 ml of medium was retrieved and discarded, the thin film of medium was left behind on the bottom of the dish overlaid with 3 ml of FC40, and a grid or individual chambers were made using the printer essentially as described previously (*9*).

The reference plate used in Fig. 4 is a custom-made product supplied by iotaSciences; it consists of a glass support with a series of unique identifiers printed on it in a 16 × 16 matrix (i.e., A1 to A16 on the first row and P1 to P16 on the last row; 1.9-mm spacing between identifiers).

### Imaging

Images of dishes were taken using a digital single-lens reflex (SLR) camera (Nikon D610). Bright-field, phase-contrast, and fluorescence microscopy images of grids with and without cells were collected using a zoom lens and digital SLR camera (Nikon D7100 DSLR) connected to an epifluorescent microscope (Olympus IX53; 1.25×, 4×, 10×, and 25× objectives) with translation stage and overhead illuminator (Olympus IX3 with filters). Images in Figs. 1 (B and C) and 3B had brightness increased by 20%. For the fluorescence images in Fig. 5B, contrast and brightness were enhanced using ImageJ, and for Fig. 6 (B and C) and fig. S4B, contrast was increased by 40%.

Images of wounds used for the graph in fig. S3 were collected daily, and post-processing was performed using ImageJ. Areas free of cells (wounds) were manually selected and measured.

Images of colonies used for fig. S2 were taken after 8 days from plating. The outline of each colony was selected manually, and the area of its footprint was measured using ImageJ.

### Cost

FC40 is the only reagent required by the technique in addition to those already used in conventional cell culture. On the basis of the price for FC40 (bottle of 2.7 liters; Acota, product no. 51142-49-5), and each dish receiving 2 ml, the FC40 cost/dish is £0.4. Fluidic chambers use volumes up to 100- to 1000-fold smaller than 96-well plates, resulting in linear reduction in the cost of reagents required. Furthermore, the excellent optics of the chambers have positive effects on medium costs during cloning. Thus, users can reliably detect chambers containing only one cell immediately after plating; this is impossible in a microplate well, as the solid walls obscure views of any cells abutting walls (this is why many perform a second round of cloning to increase the chances that a “clone” is truly derived from only one cell). Moreover, clones can be picked sooner [e.g., after 8 days in the examples shown in Fig. 3, A (b) and B (b)], compared to the ~14 days or more usually taken using a microplate well (again, the excellent optics enable users to detect the whole clone). In terms of equipment, the printer (a three-axis traverse plus syringe pump) can vary in cost depending on the specifications. In our case, this was provided by iotaSciences as an early prototype research tool for development purposes.

## Supplementary Material

http://advances.sciencemag.org/cgi/content/full/5/6/eaav8002/DC1

Download PDF

Movie S1

Movie S2

## SUPPLEMENTARY MATERIALS

Supplementary material for this article is available at http://advances.sciencemag.org/cgi/content/full/5/6/eaav8002/DC1 Fig. S1. Portraits made on 6-cm dishes. Fig. S2. Growth of clones in chambers in grids compared to wells in microplates. Fig. S3. Wound-healing rates (HEK cells). Fig. S4. A wound-healing assay where Matrigel is added after wounding. Movie S1. Reconfiguring patterns. Movie S2. Creating chambers for the isolation of clones.
