# Supplementary material for: Raising fluid walls around living cells
Source: Sci Adv. 2019 Jun 5;5(6):eaav8002. doi: 10.1126/sciadv.aav8002 (PMC6551168; doi:10.1126/sciadv.aav8002)
Supplement: http://advances.sciencemag.org/cgi/content/full/5/6/eaav8002/DC1 [file supp_5_6_eaav8002__index.html]

Science Advances | Science Advances

## Supplementary Materials

**The PDF file includes:**

- Fig. S1. Portraits made on 6-cm dishes.
- Fig. S2. Growth of clones in chambers in grids compared to wells in microplates.
- Fig. S3. Wound-healing rates (HEK cells).
- Fig. S4. A wound-healing assay where Matrigel is added after wounding.
- Legends for movies S1 and S2

Download PDF

**Other Supplementary Material for this manuscript includes the following:**

- Movie S1 (.mp4 format). Reconfiguring patterns.
- Movie S2 (.mp4 format). Creating chambers for the isolation of clones.

**Files in this Data Supplement:**

- Adobe PDF - aav8002\_SM.pdf
