# Supplementary material for: Raising fluid walls around living cells
Source: Sci Adv. 2019 Jun 5;5(6):eaav8002. doi: 10.1126/sciadv.aav8002 (PMC6551168; doi:10.1126/sciadv.aav8002)
Supplement: Download PDF [file aav8002_SM.pdf]

Science Advances
AAAS

advances.sciencemag.org/cgi/content/full/5/6/eaav8002/DC1

Supplementary Materials for

Raising fluid walls around living cells

Cristian Soitu, Alexander Feuerborn, Cyril Deroy, Alfonso A. Castrejón-Pita, Peter R. Cook, Edmond J. Walsh*

*Corresponding author. Email: edmond.walsh@eng.ox.ac.uk

Published 5 June 2019, *Sci. Adv.* **5**, eaav8002 (2018)
DOI: 10.1126/sciadv.aav8002

The PDF file includes:

Fig. S1. Portraits made on 6-cm dishes.
Fig. S2. Growth of clones in chambers in grids compared to wells in microplates.
Fig. S3. Wound-healing rates (HEK cells).
Fig. S4. A wound-healing assay where Matrigel is added after wounding.
Legends for movies S1 and S2

Other Supplementary Material for this manuscript includes the following:

(available at advances.sciencemag.org/cgi/content/full/5/6/eaav8002/DC1)

Movie S1 (.mp4 format). Reconfiguring patterns.
Movie S2 (.mp4 format). Creating chambers for the isolation of clones.

Supplementary Materials

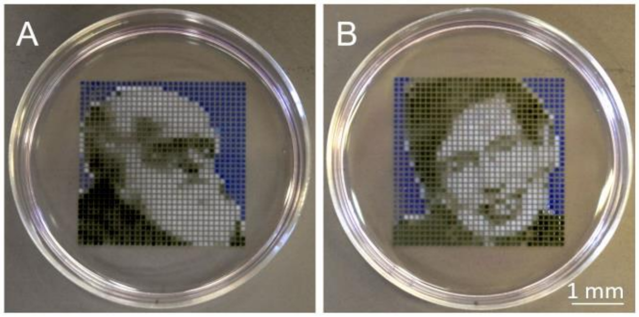

Fig. S1. Portraits made on 6-cm dishes. Grids (32 x 32 arrays; 1 mm spacing) were made, and a palette of 16 colors (blue and 15 shades of grey) prepared on a glass slide designed to hold 16 droplets. In Matlab, initial portraits have been converted to grayscale images, and their resolution was changed to match the size of the grids (i.e., 32 x 32 pixels). Intensities of individual pixels in the image were distributed into 15 divisions, each corresponding to a shade of grey. The background around heads was cropped and allocated the color blue. Volumes of 60 nl of blue dye, or one of the 15 shades of grey dye, were then added to corresponding chambers to reproduce portraits of (**A**) Charles Darwin and (**B**) Stephen Hawking. (Photo Credit: Cristian Soitu, University of Oxford).

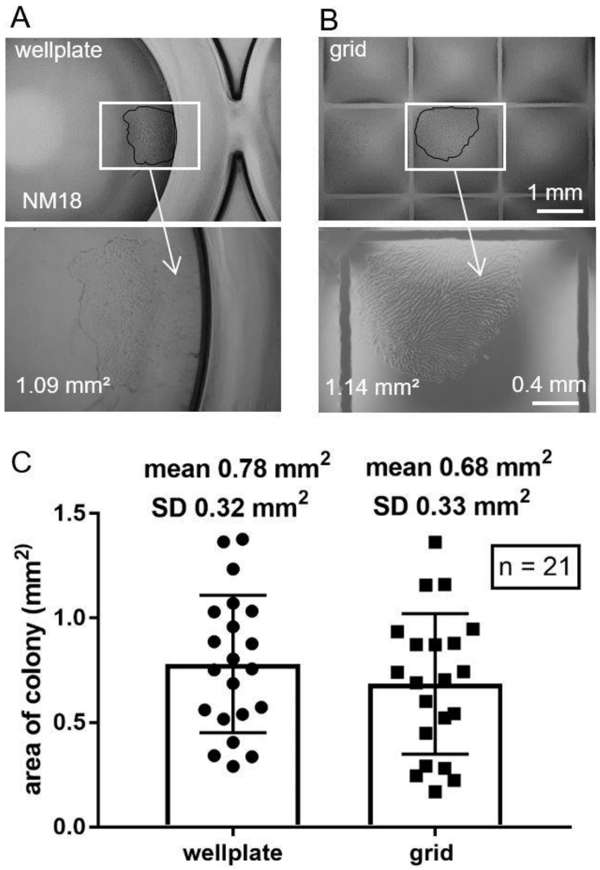

Fig. S2. Growth of clones in chambers in grids compared to wells in microplates. Single NM18 cells were plated either in chambers in 96-well plates, or in chambers in 16x16 grids; after growth for 8 days, images of clones were collected, and areas of clones (a measure of growth) determined using ImageJ. (**A** and **B**) Typical images. Top: images of parts of wells/chambers (black lines mark colony edges). Bottom: areas indicated above are shown at higher magnification (numbers at bottom left give clone areas). (**C**) Areas of colonies grown in wells and chambers are similar (p value = 0.358, unpaired t-test). One would expect a clone in a chamber (which has 1/8th the area of a well) to be slightly smaller due to the effects of contact inhibition (as cells stop growing when the reach the edge of the available area). (Photo Credit: Cristian Soitu, University of Oxford).

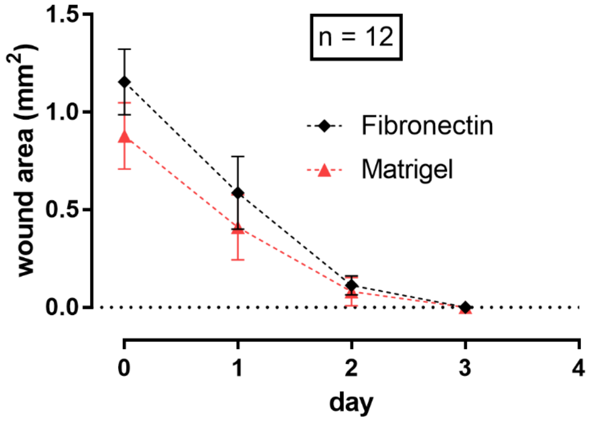

Fig. S3. Wound-healing rates (HEK cells). Using images collected as in Fig. 6, the area of unfilled wound at different times was determined using ImageJ. Although wounds were created using the same Teflon rod, wound area on fibronectin-coated dishes is initially larger than on Matrigel-coated dishes because cells on fibronectin have stronger cell:cell interactions and more tend to be removed from the surface as the rod passes. Both wounds heal completely after 3 days. The difference in healing rates between wounds on fibronectin (mean 0.384 mm^2/day, SD 0.056 mm^2/day) and those on Matrigel (mean 0.292 mm^2/day, SD 0.056 mm^2/day) is significant (p value = 0.0006, unpaired t-test).

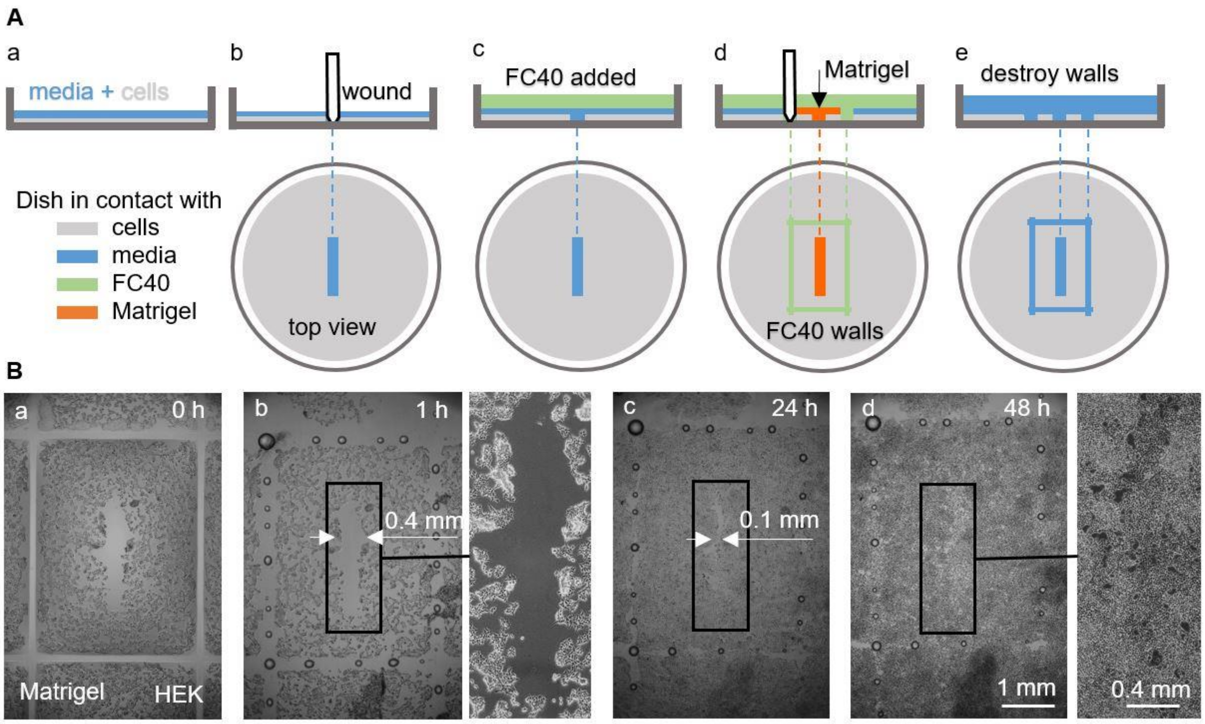

Fig. S4. A wound-healing assay where Matrigel is added after wounding. The workflow in Fig. 6 involved coating the surface before plating cells and wounding; here, the coating is added after plating and wounding (and cells begin their migration into the wound in the presence of Matrigel in the medium).

A. Cartoon illustrating the workflow. (**a**) 600,000 HEKs were plated on a dish and grown for 24 h to confluency. (**b**) The stylus is scraped across the surface to remove cells in its path and create a wound (0.4 x 2 mm). (**c**) Most medium is now removed manually to leave a thin film in the dish, and FC40 (3 ml) added (again manually). (**d**) A rectangular chamber (3 x 4 mm) is built around the wound to isolate it from the rest of the dish, and Matrigel added to this chamber (2 µl; final concentration of 1 µg/cm^2; cells are incubated for 1 h). In an experiment, a number of wounds would be created in one dish, chambers built around them, and individual chambers filled with different agents so that their effects can be compared. (**e**) Fluid walls are now destroyed (FC40 is removed manually, and the dish washed thoroughly with 3 ml medium to remove unattached Matrigel); wound healing is monitored by microscopy.

B. Images of wounds at different times. (**a**) The wounded region immediately after building the surrounding wall (before adding Matrigel). (**b**) The same region after incubation (1 h) in Matrigel, wall destruction, and removal of unattached Matrigel (FC40 is removed manually, and the dish washed thoroughly with 3 ml medium); traces of FC40 remain. (**c**) After further incubation (24 h), cells have grown into the wound (wound width is $<$0.1 mm). Healing occurs slightly faster than in Fig. 6. (**d**) After 48 h, wounds heal completely.

Movies

Movie S1. Reconfiguring patterns. An initial pattern (a circle within a triangle within a square with 9 mm sides) is printed, and different dyes added to each enclosure. The three dyes do not mix, being separated by FC40 walls. Walls around the two inner compartments are destroyed successively by pumping a large volume (55 µl) of yellow dye into the centre. Most of the fluid (60 µl) is now retrieved from the square, and a new pattern created (a triangle within a circle within the surviving square). Dyes are now added to the different compartments as before. The movie plays at 3x speed.

Movie S2. Creating chambers for the isolation of clones. Sets of three chambers of various shapes – circle, square (sides 2 mm), and triangle – are created, and red dye (1 µl) is added to each chamber. The movie plays in real time as the first two patterns are printed, and then at 3x the speed.
